# Cholangioscopy-guided laser ablation for intraductal papillary neoplasm of bile duct

**DOI:** 10.1055/a-2357-2408

**Published:** 2024-07-26

**Authors:** Yuhuan Wu, Weigang Gu, Ka Shing Cheung, Jianfeng Yang, Xiaofeng Zhang, Hangbin Jin

**Affiliations:** 1 74630 Zhejiang Chinese Medical University The Fourth School of Clinical Medicine, Hangzhou, China; 2 Gastroenterology, Affiliated Hangzhou First People’s Hospital, School of Medicine, Westlake University, Hangzhou, China; 3 Key Laboratory of Integrated Traditional Chinese and Western Medicine for Biliary and Pancreatic Diseases of Zhejiang Province, Hangzhou, China; 4 Hangzhou Institute of Digestive Diseases, Hangzhou, China; 5 444333 Medicine, The University of Hong Kong-Shenzhen Hospital, Shenzhen, China; 6 Medicine, School of Clinical Medicine, Queen Mary Hospital, The University of Hong Kong, Hong Kong; 7 Key Laboratory of Integrated Traditional Chinese and Western Medicine for Biliary and Pancreatic Diseases of Zhejiang Province, Hangzhou, China; 8 Key Laboratory of Integrated Traditional Chinese and Western Medicine for Biliary and Pancreatic Diseases of Zhejiang Province, Hangzhou, China

A 65-year-old man with a history of metastatic bladder cancer was admitted with jaundice. Blood tests revealed a cholestatic pattern of liver function, hyperbilirubinemia, and deranged clotting profile. Computed tomography and magnetic resonance cholangiopancreatography showed cirrhosis and dilated extrahepatic and intrahepatic biliary systems without hyperdense stones ( Fig. 1 ). Endoscopic retrograde cholangiopancreatography revealed a fish-mouth deformity of the papillary opening ( Fig. 2 ), dilated intrahepatic ducts, and poor contrast filling of the common bile duct ( Fig. 3 ). Repeated balloon trawling (18 mm) yielded a copious amount of jelly-like mucus. Cholangioscopy (SpyGlass; Boston Scientific, Natick, Massachusetts, United States) revealed multiple foci of papillary growth in the upper common bile duct, common hepatic duct, and proximal left biliary duct ( Fig. 4 ). The biopsy samples revealed papillary proliferation with a gastric subtype and low-grade dysplasia. A diagnosis of intraductal papillary neoplasm of the bile duct (IPNB) was made. Repeat cholangioscopy was performed by introducing an end-on irradiation fiber connected to a laser system (Leonardo 1470 nm/980 nm dual-wavelength laser; CeramOptec GmbH/Biolitec AG, Bonn, Germany) ( Fig. 5 ). Ablation of the papillary growth was performed until a whitish discoloration and necrosis appeared ( Video 1 ). The patient did not experience any discomfort or adverse events after the procedure and was discharged 9 days later.

**Fig. 1:**
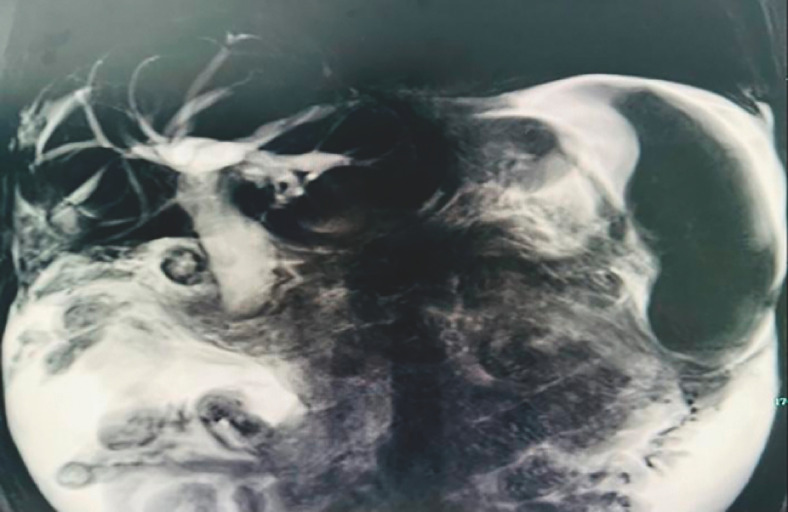
Magnetic resonance cholangiopancreatography revealing dilated extrahepatic and intrahepatic biliary systems.

**Fig. 2:**
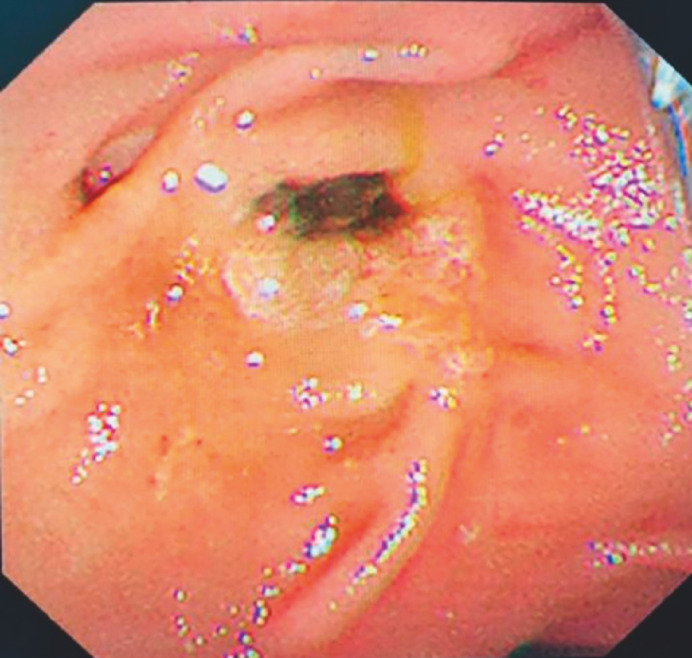
Endoscopic image of fish-mouth deformity of the papilla.

**Fig. 3:**
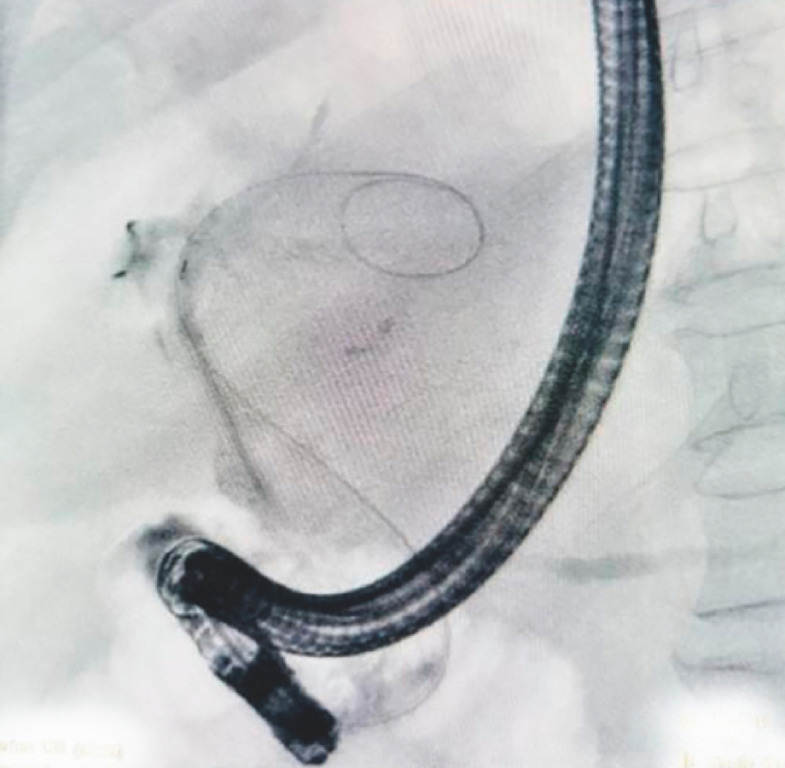
Endoscopic retrograde cholangiopancreatoscopy revealing filling defects due to mucus.

**Fig. 4:**
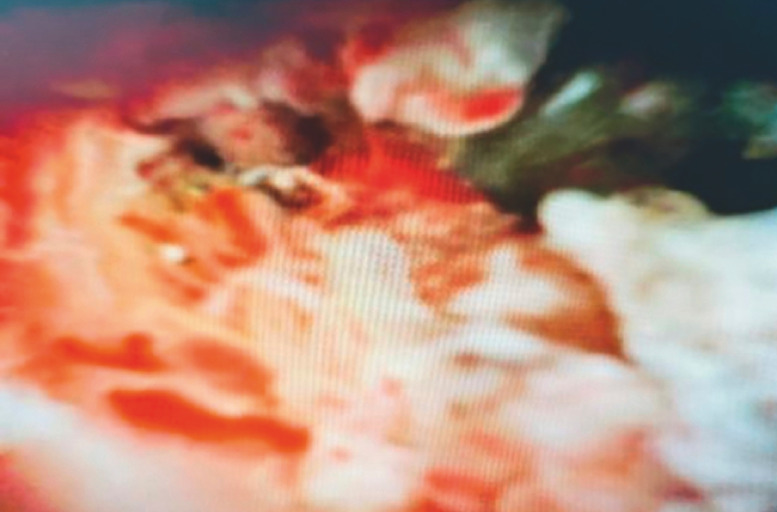
Cholangioscopic image revealing papillary growths.

**Fig. 5:**
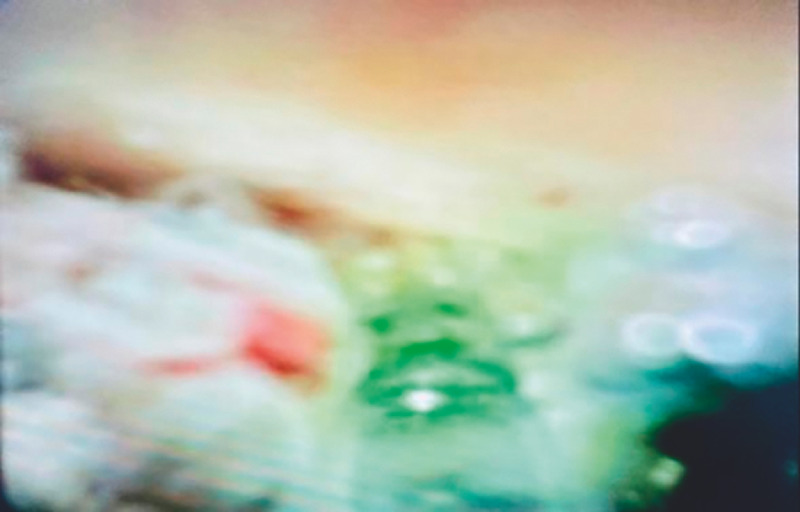
Application of cholangioscopy-guided laser on the papillary growth.

Cholangioscopy-guided laser ablation of intraductal papillary mucinous neoplasm.Video 1

IPNB is an epithelial tumor characterized by intraductal papillary proliferation and a thin fibrovascular stem on histological analysis 1 . Due to the risk of progression to cancer, surgical resection is recommended. However, surgery was contraindicated for our patient. Furthermore, the insertion of a biliary stent does not relieve biliary obstruction due to the viscous nature of the mucus. The use of cholangioscopy-guided laser to dissect benign biliopancreatic ductal strictures 2 3 and for ablation of cholangiocarcinoma 4 has been reported previously. Our experience suggests that laser has good ablative effects and may be a promising treatment modality for IPNB, particularly for patients who are unfit for surgery.

Endoscopy_UCTN_Code_TTT_1AR_2AF

## Notes

Endoscopy E-Videos https://eref.thieme.de/e-videos
*E-Videos* is an open access online section of the journal *Endoscopy* , reporting on interesting cases and new techniques in gastroenterological endoscopy. All papers include a high-quality video and are published with a Creative Commons CC-BY license. Endoscopy E-Videos qualify for HINARI discounts and waivers and eligibility is automatically checked during the submission process. We grant 100% waivers to articles whose corresponding authors are based in Group A countries and 50% waivers to those who are based in Group B countries as classified by Research4Life (see: https://www.research4life.org/access/eligibility/ ). This section has its own submission website at https://mc.manuscriptcentral.com/e-videos .

## References

[1] Sakai Y Ohtsuka M Sugiyama H Current status of diagnosis and therapy for intraductal papillary neoplasm of the bile duct World J Gastroenterol 2021 27 1569 1577 10.3748/wjg.v27.i15.1569 33958844 PMC8058653

[2] Mittal C Shah RJ Pancreatoscopy-guided laser dissection and ablation for treatment of benign and neoplastic pancreatic disorders: an initial report (with videos) Gastrointest Endosc 2019 89 384 389 10.1016/j.gie.2018.08.045 30176224

[3] Han S Shah RJ Cholangiopancreatoscopy-guided laser dissection and ablation for pancreas and biliary strictures and neoplasia Endosc Int Open 2020 8 E1091 E1096 10.1055/a-1192-4082 32743063 PMC7373658

[4] Xia M Hu X Zhang T Laser ablation under intraductal cholangioscopic guidance for cholangiocarcinoma Endoscopy 2023 55 E590 E591 10.1055/a-2051-7984 36996886 PMC10063346

